# *N*-(2,3-Dimethyl­phen­yl)acetamide

**DOI:** 10.1107/S1600536809011891

**Published:** 2009-04-08

**Authors:** B. Thimme Gowda, Sabine Foro, Hiromitsu Terao, Hartmut Fuess

**Affiliations:** a Department of Chemistry, Mangalore University, Mangalagangotri 574 199, Mangalore, India; b Institute of Materials Science, Darmstadt University of Technology, Petersenstrasse 23, D-64287 Darmstadt, Germany; c Faculty of Integrated Arts and Sciences, Tokushima University, Minamijosanjima-cho, Tokushima 770-8502, Japan

## Abstract

The conformation of the N—H bond in the structure of the title compound, C_10_H_13_NO, is *syn* to both the 2- and 3-methyl substituents on the aromatic ring, and is *anti* to the C=O bond. N—H⋯O hydrogen bonds link the mol­ecules into supra­molecular chains.

## Related literature

For preparation of the compound, see: Gowda *et al.* (2006 ▶). For related structures, see: Gowda *et al.* (2007*a* ▶,*b* ▶; 2008 ▶)
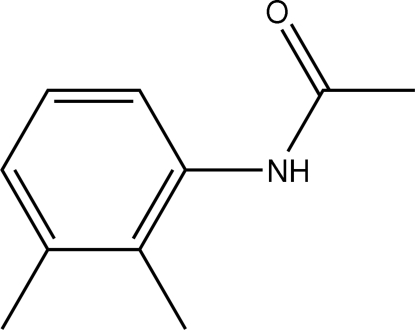


## Experimental

### 

#### Crystal data

C_10_H_13_NO
*M* *_r_* = 163.21
Monoclinic, 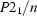

*a* = 4.7961 (5) Å
*b* = 12.385 (1) Å
*c* = 15.475 (2) Å
β = 96.23 (1)°
*V* = 913.78 (17) Å^3^
*Z* = 4
Mo *K*α radiation
μ = 0.08 mm^−1^
*T* = 299 K
0.45 × 0.08 × 0.04 mm

#### Data collection

Oxford Diffraction Xcalibur diffractometer with a Sapphire CCD detector
Absorption correction: multi-scan (*CrysAlis RED*; Oxford Diffraction, 2007 ▶) *T*_min_ = 0.967, *T*_max_ = 0.993
5890 measured reflections
1660 independent reflections
1121 reflections with *I* > 2σ(*I*)
*R*_int_ = 0.035

#### Refinement

*R*[*F* ^2^ > 2σ(*F* ^2^)] = 0.073
*wR*(*F* ^2^) = 0.156
*S* = 1.26
1660 reflections
115 parameters
H atoms treated by a mixture of independent and constrained refinement
Δρ_max_ = 0.23 e Å^−3^
Δρ_min_ = −0.18 e Å^−3^

### 

Data collection: *CrysAlis CCD* (Oxford Diffraction, 2004 ▶); cell refinement: *CrysAlis RED* (Oxford Diffraction, 2007 ▶); data reduction: *CrysAlis RED*; program(s) used to solve structure: *SHELXS97* (Sheldrick, 2008 ▶); program(s) used to refine structure: *SHELXL97* (Sheldrick, 2008 ▶); molecular graphics: *PLATON* (Spek, 2009 ▶); software used to prepare material for publication: *SHELXL97*.

## Supplementary Material

Crystal structure: contains datablocks I, global. DOI: 10.1107/S1600536809011891/tk2409sup1.cif

Structure factors: contains datablocks I. DOI: 10.1107/S1600536809011891/tk2409Isup2.hkl

Additional supplementary materials:  crystallographic information; 3D view; checkCIF report

## Figures and Tables

**Table 1:** Hydrogen-bond geometry (Å, °)

*D*—H⋯*A*	*D*—H	H⋯*A*	*D*⋯*A*	*D*—H⋯*A*
N1—H1*N*⋯O1^i^	0.85 (3)	2.06 (3)	2.901 (3)	169 (3)

Symmetry code: (i) 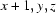.

**Fig. 1.**
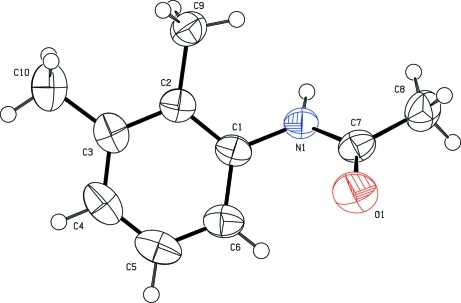
Molecular structure of (I), showing the atom labeling scheme and displacement ellipsoids are drawn at the 50% probability level.

**Fig. 2.**
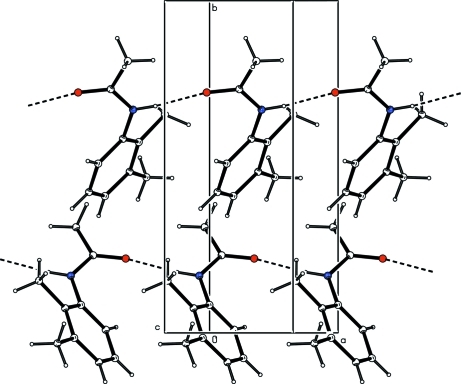
Molecular packing of (I) with hydrogen bonding shown as dashed lines.

## supplementary crystallographic information

### Comment

As a part of studying the ring- and side-chain substitutions on the crystal structures of chemically and biologically important class of compounds such as acetanilides (Gowda *et al.*, 2007*a*,*b*; 2008), we report herein the crystal structure of *N*-(2,3-dimethylphenyl)acetamide, (I). The conformation of the C=O bond is *anti* to the N—H bond, Fig. 1. The conformation of the N—H bond is *syn* to both the 2- and 3-methyl substituents in the aromatic ring, similar to that observed with respect to to the 2- and 3-chloro substituents in *N*-(2,3-dichlorophenyl)acetamide (Gowda *et al.*, 2007*a*), but in contrast to the *anti* conformation observed with respect to the 2-methyl group in *N*-(2-methylphenyl)acetamide (Gowda *et al.*, 2007*b*). The molecules in (I) are linked into supramolecular chains along the a axis through intermolecular N1—H1···O1 hydrogen bonding (Table 1) as shown in Fig. 2.

### Experimental

Compound (I) was prepared according to the literature method (Gowda *et al.*, 2006) and crystals were obtained from its ethanol solution held at room temperature.

### Refinement

The N-bound H atom was located in difference map, and refined with N—H = 0.85 (3) Å. The remaining H atoms were positioned with in their idealized geometry using a riding model with C—H = 0.93–0.96 Å, and with U_iso_(H) set to 1.2 x *U*_eq_(C).

### Crystal data

**Table:**

C_10_H_13_NO	*F*(000) = 352
*M**_r_* = 163.21	*D*_x_ = 1.186 Mg m^−^^3^
Monoclinic, *P*2_1_/*n*	Mo *K*α radiation, λ = 0.71073 Å
Hall symbol: -P 2yn	Cell parameters from 2448 reflections
*a* = 4.7961 (5) Å	θ = 2.6–27.6°
*b* = 12.385 (1) Å	µ = 0.08 mm^−^^1^
*c* = 15.475 (2) Å	*T* = 299 K
β = 96.23 (1)°	Needle, colourless
*V* = 913.78 (17) Å^3^	0.45 × 0.08 × 0.04 mm
*Z* = 4	

### Data collection

**Table:**

Oxford Diffraction Xcalibur diffractometer with a Sapphire CCD detector	1660 independent reflections
Radiation source: fine-focus sealed tube	1121 reflections with *I* > 2σ(*I*)
graphite	*R*_int_ = 0.035
Rotation method data acquisition using ω and phi scans.	θ_max_ = 25.3°, θ_min_ = 2.7°
Absorption correction: multi-scan (*CrysAlis RED*; Oxford Diffraction, 2007)	*h* = −3→5
*T*_min_ = 0.967, *T*_max_ = 0.993	*k* = −14→13
5890 measured reflections	*l* = −18→18

### Refinement

**Table:**

Refinement on *F*^2^	Primary atom site location: structure-invariant direct methods
Least-squares matrix: full	Secondary atom site location: difference Fourier map
*R*[*F*^2^ > 2σ(*F*^2^)] = 0.073	Hydrogen site location: inferred from neighbouring sites
*wR*(*F*^2^) = 0.156	H atoms treated by a mixture of independent and constrained refinement
*S* = 1.26	*w* = 1/[σ^2^(*F*_o_^2^) + (0.0286*P*)^2^ + 0.8009*P*] where *P* = (*F*_o_^2^ + 2*F*_c_^2^)/3
1660 reflections	(Δ/σ)_max_ = 0.001
115 parameters	Δρ_max_ = 0.23 e Å^−^^3^
0 restraints	Δρ_min_ = −0.18 e Å^−^^3^

### Special details

**Table:**

Experimental. Absorption correction details: CrysAlis RED, Oxford Diffraction Ltd., 2007 Empirical absorption correction using spherical harmonics, implemented in SCALE3 ABSPACK scaling algorithm.
Geometry. All e.s.d.'s (except the e.s.d. in the dihedral angle between two l.s. planes) are estimated using the full covariance matrix. The cell e.s.d.'s are taken into account individually in the estimation of e.s.d.'s in distances, angles and torsion angles; correlations between e.s.d.'s in cell parameters are only used when they are defined by crystal symmetry. An approximate (isotropic) treatment of cell e.s.d.'s is used for estimating e.s.d.'s involving l.s. planes.
Refinement. Refinement of *F*^2^ against ALL reflections. The weighted *R*-factor *wR* and goodness of fit *S* are based on *F*^2^, conventional *R*-factors *R* are based on *F*, with *F* set to zero for negative *F*^2^. The threshold expression of *F*^2^ > σ(*F*^2^) is used only for calculating *R*-factors(gt) *etc*. and is not relevant to the choice of reflections for refinement. *R*-factors based on *F*^2^ are statistically about twice as large as those based on *F*, and *R*- factors based on ALL data will be even larger.

### Fractional atomic coordinates and isotropic or equivalent isotropic displacement parameters (Å^2^)

**Table:**

	*x*	*y*	*z*	*U*_iso_*/*U*_eq_	
C1	−0.0442 (6)	0.9157 (3)	0.87038 (19)	0.0414 (8)	
C2	0.0495 (6)	0.9258 (3)	0.7884 (2)	0.0424 (8)	
C3	−0.0385 (6)	1.0149 (3)	0.7370 (2)	0.0502 (9)	
C4	−0.2211 (7)	1.0883 (3)	0.7678 (3)	0.0620 (10)	
H4	−0.2811	1.1473	0.7335	0.074*	
C5	−0.3162 (7)	1.0761 (3)	0.8479 (3)	0.0639 (11)	
H5	−0.4413	1.1260	0.8668	0.077*	
C6	−0.2269 (6)	0.9905 (3)	0.9001 (2)	0.0521 (9)	
H6	−0.2881	0.9828	0.9548	0.063*	
C7	−0.1038 (6)	0.7664 (3)	0.9715 (2)	0.0468 (8)	
C8	0.0486 (7)	0.6820 (3)	1.0266 (2)	0.0606 (10)	
H8A	0.0383	0.6984	1.0868	0.073*	
H8B	0.2414	0.6803	1.0153	0.073*	
H8C	−0.0357	0.6128	1.0132	0.073*	
C9	0.2419 (7)	0.8429 (3)	0.7556 (2)	0.0523 (9)	
H9A	0.2524	0.7811	0.7932	0.063*	
H9B	0.4256	0.8734	0.7548	0.063*	
H9C	0.1703	0.8214	0.6978	0.063*	
C10	0.0608 (8)	1.0314 (3)	0.6489 (2)	0.0690 (11)	
H10A	−0.0029	0.9726	0.6114	0.083*	
H10B	0.2620	1.0341	0.6547	0.083*	
H10C	−0.0134	1.0980	0.6244	0.083*	
N1	0.0539 (5)	0.8282 (2)	0.92501 (16)	0.0432 (7)	
H1N	0.230 (7)	0.818 (3)	0.931 (2)	0.052*	
O1	−0.3582 (4)	0.7763 (2)	0.96925 (18)	0.0722 (8)	

### Atomic displacement parameters (Å^2^)

**Table:**

	*U*^11^	*U*^22^	*U*^33^	*U*^12^	*U*^13^	*U*^23^
C1	0.0283 (15)	0.045 (2)	0.0503 (19)	0.0009 (14)	−0.0004 (13)	−0.0039 (15)
C2	0.0317 (16)	0.045 (2)	0.0497 (19)	−0.0051 (14)	0.0012 (13)	−0.0040 (15)
C3	0.0429 (18)	0.047 (2)	0.058 (2)	−0.0068 (16)	−0.0060 (15)	0.0043 (17)
C4	0.054 (2)	0.046 (2)	0.082 (3)	0.0039 (18)	−0.010 (2)	0.008 (2)
C5	0.050 (2)	0.057 (3)	0.083 (3)	0.0135 (18)	−0.0006 (19)	−0.012 (2)
C6	0.0428 (18)	0.057 (2)	0.056 (2)	0.0048 (17)	0.0034 (15)	−0.0088 (18)
C7	0.0351 (18)	0.056 (2)	0.0500 (19)	−0.0013 (16)	0.0092 (14)	−0.0032 (17)
C8	0.048 (2)	0.071 (3)	0.065 (2)	−0.0048 (18)	0.0144 (17)	0.015 (2)
C9	0.0497 (19)	0.059 (2)	0.0499 (19)	0.0009 (17)	0.0121 (15)	0.0004 (17)
C10	0.074 (3)	0.071 (3)	0.060 (2)	−0.009 (2)	−0.0039 (19)	0.014 (2)
N1	0.0272 (13)	0.0530 (17)	0.0504 (15)	0.0043 (13)	0.0084 (12)	0.0046 (14)
O1	0.0289 (12)	0.085 (2)	0.104 (2)	0.0005 (12)	0.0148 (12)	0.0147 (16)

### Geometric parameters (Å, °)

**Table:**

C1—C6	1.388 (4)	C7—N1	1.339 (4)
C1—C2	1.396 (4)	C7—C8	1.490 (5)
C1—N1	1.423 (4)	C8—H8A	0.9600
C2—C3	1.399 (4)	C8—H8B	0.9600
C2—C9	1.504 (4)	C8—H8C	0.9600
C3—C4	1.382 (5)	C9—H9A	0.9600
C3—C10	1.506 (5)	C9—H9B	0.9600
C4—C5	1.374 (5)	C9—H9C	0.9600
C4—H4	0.9300	C10—H10A	0.9600
C5—C6	1.373 (5)	C10—H10B	0.9600
C5—H5	0.9300	C10—H10C	0.9600
C6—H6	0.9300	N1—H1N	0.85 (3)
C7—O1	1.223 (3)		
			
C6—C1—C2	121.2 (3)	C7—C8—H8A	109.5
C6—C1—N1	119.5 (3)	C7—C8—H8B	109.5
C2—C1—N1	119.3 (3)	H8A—C8—H8B	109.5
C1—C2—C3	118.7 (3)	C7—C8—H8C	109.5
C1—C2—C9	121.0 (3)	H8A—C8—H8C	109.5
C3—C2—C9	120.3 (3)	H8B—C8—H8C	109.5
C4—C3—C2	119.1 (3)	C2—C9—H9A	109.5
C4—C3—C10	119.8 (3)	C2—C9—H9B	109.5
C2—C3—C10	121.1 (3)	H9A—C9—H9B	109.5
C5—C4—C3	121.6 (3)	C2—C9—H9C	109.5
C5—C4—H4	119.2	H9A—C9—H9C	109.5
C3—C4—H4	119.2	H9B—C9—H9C	109.5
C6—C5—C4	120.2 (3)	C3—C10—H10A	109.5
C6—C5—H5	119.9	C3—C10—H10B	109.5
C4—C5—H5	119.9	H10A—C10—H10B	109.5
C5—C6—C1	119.3 (3)	C3—C10—H10C	109.5
C5—C6—H6	120.4	H10A—C10—H10C	109.5
C1—C6—H6	120.4	H10B—C10—H10C	109.5
O1—C7—N1	123.2 (3)	C7—N1—C1	125.8 (3)
O1—C7—C8	120.9 (3)	C7—N1—H1N	117 (2)
N1—C7—C8	116.0 (3)	C1—N1—H1N	117 (2)
			
C6—C1—C2—C3	1.7 (4)	C10—C3—C4—C5	−179.9 (3)
N1—C1—C2—C3	−177.1 (3)	C3—C4—C5—C6	1.0 (5)
C6—C1—C2—C9	−178.5 (3)	C4—C5—C6—C1	−1.2 (5)
N1—C1—C2—C9	2.8 (4)	C2—C1—C6—C5	−0.2 (5)
C1—C2—C3—C4	−1.8 (4)	N1—C1—C6—C5	178.6 (3)
C9—C2—C3—C4	178.3 (3)	O1—C7—N1—C1	3.0 (5)
C1—C2—C3—C10	178.6 (3)	C8—C7—N1—C1	−177.8 (3)
C9—C2—C3—C10	−1.2 (5)	C6—C1—N1—C7	45.2 (4)
C2—C3—C4—C5	0.5 (5)	C2—C1—N1—C7	−136.0 (3)

### Hydrogen-bond geometry (Å, °)

**Table:**

*D*—H···*A*	*D*—H	H···*A*	*D*···*A*	*D*—H···*A*
N1—H1N···O1^i^	0.85 (3)	2.06 (3)	2.901 (3)	169 (3)

Symmetry codes: (i) *x*+1, *y*, *z*.
